# Diverse Malaria Presentations across National Institutes of Health South Asia International Center for Excellence in Malaria Research Sites in India

**DOI:** 10.4269/ajtmh.21-1344

**Published:** 2022-10-13

**Authors:** Rimi Chakrabarti, Laura Chery-Karschney, John White, Anjali Mascarenhas, Kristen M. Skillman, Usheer Kanjee, Prasad H. Babar, Rapatbhorn Patrapuvich, Pradyumna Kishore Mohapatra, Swati Patankar, Joseph D. Smith, Anup Anvikar, Neena Valecha, Manju Rahi, Manoj T. Duraisingh, Pradipsinh K. Rathod

**Affiliations:** ^1^ Department of Chemistry, University of Washington, Seattle, Washington;; ^2^ Department of Medicine, Goa Medical College and Hospital, Bambolim, Goa, India;; ^3^ Department of Immunology and Infectious Diseases, Harvard T. H. Chan School of Public Health, Boston, Massachusetts;; ^4^ Drug Research Unit for Malaria (DRUM), Faculty of Tropical Medicine, Mahidol University, Bangkok, Thailand;; ^5^ Division of Malaria, Regional Medical Research Center—Northeast (RMRC-NE), Dibrugarh, Assam;; ^6^ Department of Biosciences and Bioengineering, IIT Bombay, Powai, Mumbai, India;; ^7^ Seattle Children’s Research Institute, Seattle, Washington;; ^8^ National Institute of Biologicals, Noida, UP, India;; ^9^ National Institute of Malaria Research, New Delhi, India;; ^10^ Division of Epidemiology and Communicable Disease, Indian Council of Medical Research, New Delhi, India

## Abstract

The Malaria Evolution in South Asia (MESA) International Center for Excellence in Malaria Research (ICEMR) was established by the US National Institutes of Health (US NIH) as one of 10 malaria research centers in endemic countries. In 10 years of hospital-based and field-based work in India, the MESA-ICEMR has documented the changing epidemiology and transmission of malaria in four different parts of India. Malaria Evolution in South Asia-ICEMR activities, in collaboration with Indian partners, are carried out in the broad thematic areas of malaria case surveillance, vector biology and transmission, antimalarial resistance, pathogenesis, and host response. The program integrates insights from surveillance and field studies with novel basic science studies. This is a two-pronged approach determining the biology behind the disease patterns seen in the field, and generating new relevant biological questions about malaria to be tested in the field. Malaria Evolution in South Asia-ICEMR activities inform local and international stakeholders on the current status of malaria transmission in select parts of South Asia including updates on regional vectors of transmission of local parasites. The community surveys and new laboratory tools help monitor ongoing efforts to control and eliminate malaria in key regions of South Asia including the state of evolving antimalarial resistance in different parts of India, new host biomarkers of recent infection, and molecular markers of pathogenesis from uncomplicated and severe malaria.

## INTRODUCTION

The Malaria Evolution in South Asia (MESA) International Center of Excellence in Malaria Research (ICEMR) program sponsored by the US National Institutes of Health (NIH) has worked actively in India for a decade.^1^ The overall goal of this program has been to study variations in malaria parasite evolution through the lens of parasite plasticity, pathogenesis, human genetics, and vector-mediated transmission. The MESA-ICEMR has established field sites in Goa, Maharashtra, Jharkhand, and Assam that stretch across India, from the Southwest to Northeast (Figure 1A). The Goa site of the MESA-ICEMR is linked to the Medicine Department of the Goa Medical College and Hospital (GMC), the largest tertiary multispecialty health institution in the state. Goa is India’s smallest state, ∼120 km North to South and ∼80 km East to West. The GMC is centrally situated in Goa and visited by patients from across the state. The Maharashtra site is in Jawaharlal Nehru Medical College and Acharya Vinoba Bhave Rural Hospital (AVBRH) in Wardha district, located at the center of India. Like GMC, the majority of malaria cases at AVBRH are infected with *Plasmodium vivax*. Shalini Hospital, associated with Krishi Gram Vikas Kendra (KGVK), is the site in Ranchi district of Jharkhand. The most remote MESA-ICEMR site is at the Northeast corner of Assam in Dibrugarh district, within 160 km from the India–Myanmar border. It is hosted by the Regional Medical Research Center-Northeast (RMRC-NE) and has associations with Assam Medical College (AMC). The two Eastern sites KGVK and RMRC-NE see a higher proportion of *Plasmodium falciparum* infections compared with GMC and AVBRH (Figure 1B). Within India, Goa is at the low end of the transmission continuum and is classified as a Category 1 state (Elimination Phase) by the National Framework for Malaria Elimination (NFME)^2^ in India 2016–2030 and National Strategic Plan (NSP) for Malaria Elimination in India 2017–2022.^3^ Maharashtra and Assam have moderate transmission intensity and are Category 2 states (Preelimination Phase), whereas Jharkhand is one of the high-transmission states classified as Category 3 (Intensified Control Phase) (Figure 1C). Of these four sites, Goa is at the top of the Healthcare Access and Quality Index (HAX) at 64.8 (59.6–68.8) while Assam is at the other end at 34.0 (30.3–38.1).^4^

**Figure 1.**
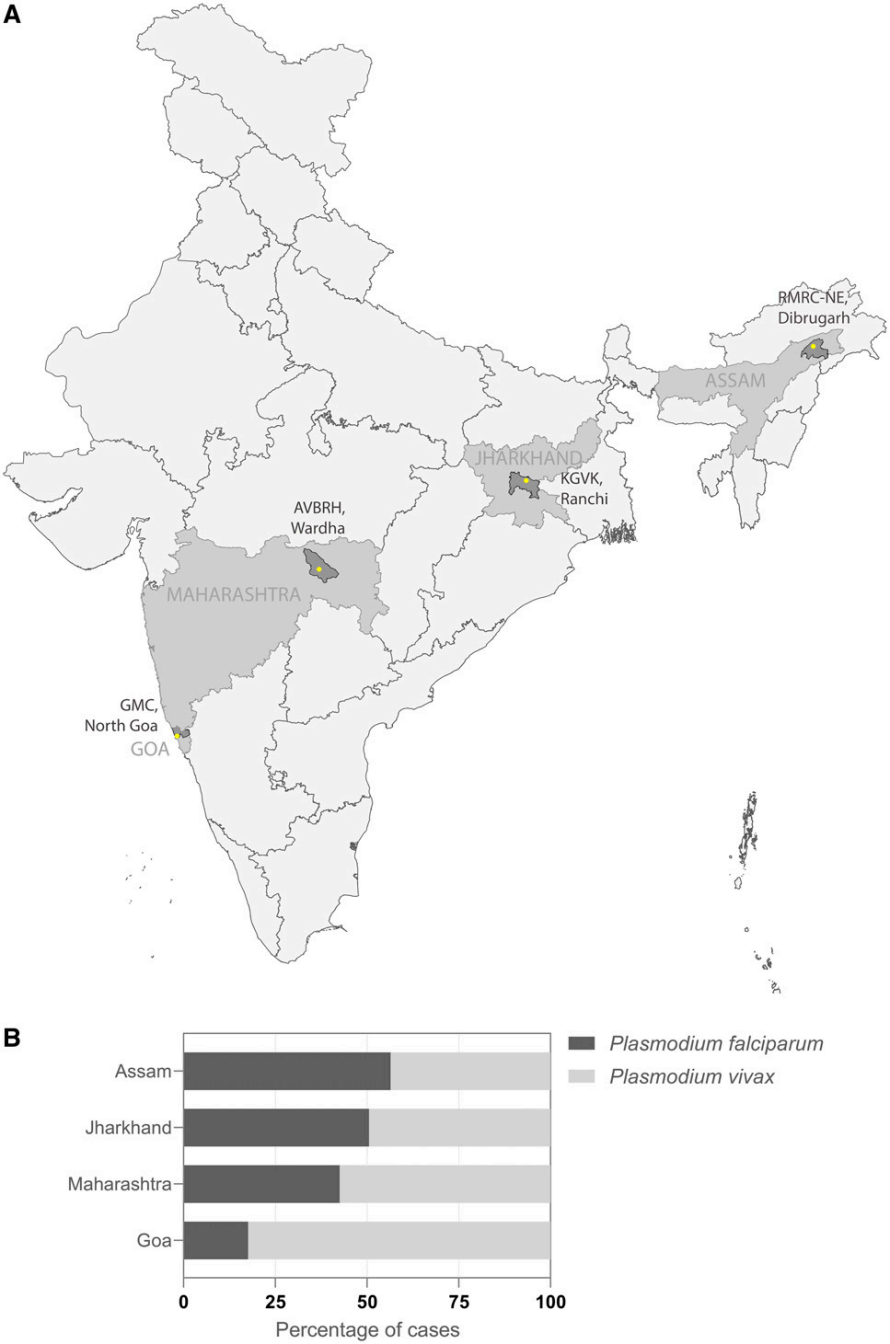
(**A**) Malaria Evolution in South Asia (MESA) International Center for Excellence in Malaria Research (ICEMR) sites in India. Location of the four sites at 1) Goa Medical College (GMC), North Goa district, Goa state, 2) Jawaharlal Nehru Medical College and Acharya Vinoba Bhave Rural Hospital (AVBRH), Wardha district, Maharashtra state, 3) Shalini Memorial Hospitals, Krishi Gram Vikas Kendra (KGVK), Ranchi district, Jharkhand state, and 4) Assam Medical College and Regional Medical Research Center—Northeast (RMRC-NE), Dibrugarh district, Assam state. The study states are marked in gray and the study districts are marked in dark gray. (**B**) *Plasmodium falciparum:P. vivax* ratio in states where the study sites are located. Cases include both uncomplicated and complicated malaria. (**C**) Categorization of Indian states on the Annual Parasite Index (API) based transmission continuum according to malaria transmission data reported by National Vector Borne Disease Control Program (NVBDCP) (now called National Center for Vector Borne Disease Control NCVBDC). Category 1 contains states with API less than one and all the districts in the state are with API less than one. Category 2 contains states having API less than one and one or more districts reporting API more than one. Category 3 contains states with API more than one. MESA site Goa is in Category 1, Assam and Maharashtra are Category 2 states while Jharkhand is a Category 3 state. GA = Goa; AS = Assam; MH = Maharashtra; JH = Jharkhand. Other state names also follow the two-letter code (alpha-2) according to the ISO 3166-1 standard (https://www.iso.org/obp/ui/#iso:code:3166:IN). * Till October 2021.

**Figure 1.**
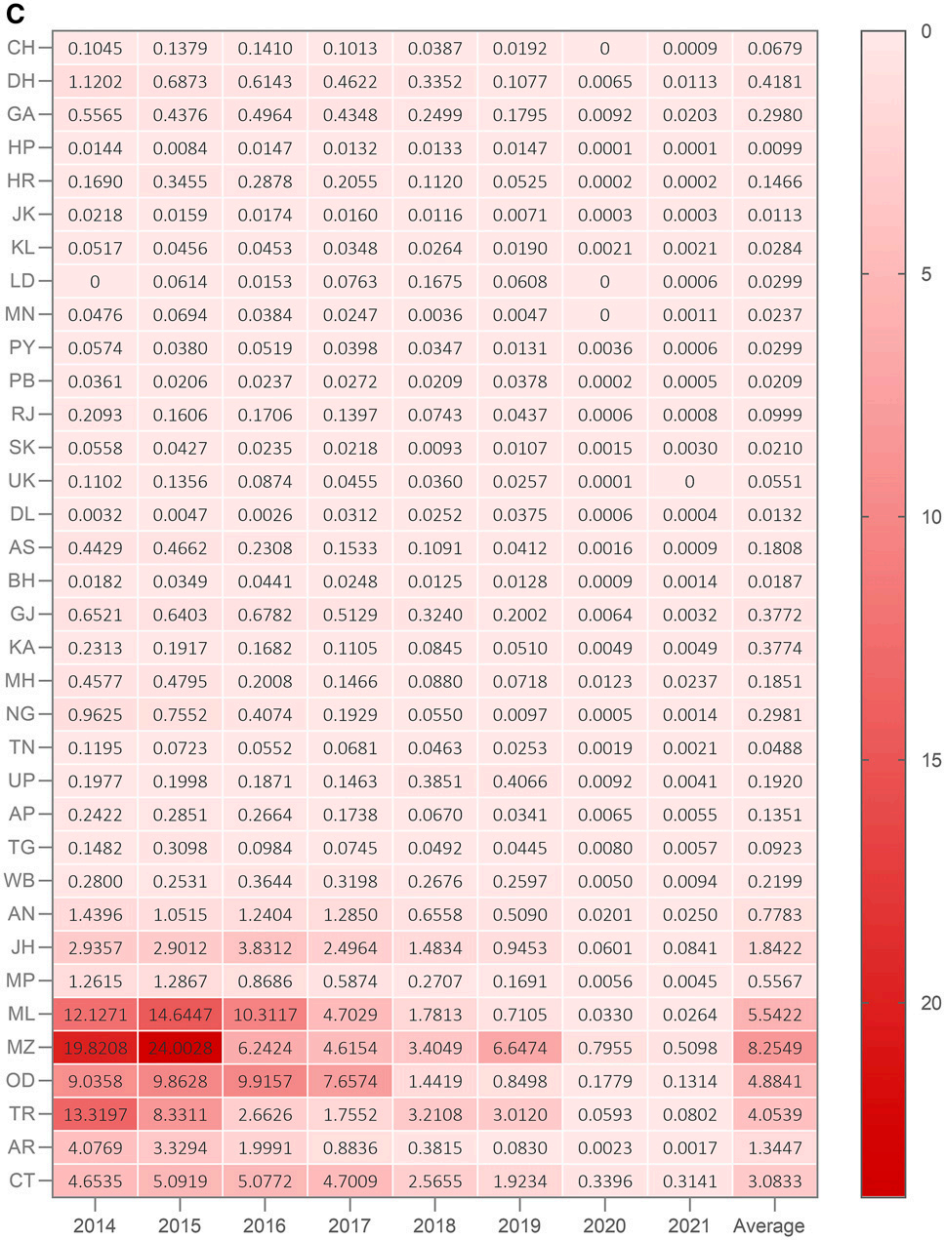
Continued

Together, the MESA-ICEMR sites are uniquely positioned to capture variables, which contribute to heterogeneity and complexity of malaria transmission in different healthcare accessibility settings in India. The sites regularly report on parasite species distribution, pathogenesis of severe and uncomplicated malaria, changes in malaria transmission, and monitor risk of importing Artemisinin Combination Therapy (ACT) resistance from Southeast Asia. The present perspective reveals the clinical and translational value of MESA-ICEMR conducted research in India, and how such research can contribute to improved malaria control strategies and its implementation. The relative impact of this research on malaria policy-making in India is discussed in an accompanying article titled “International Center of Excellence for Malaria Research for South Asia and Broader Malaria Research in India.”

## HEALTH FACILITY AND HOUSEHOLD-BASED SURVEILLANCE

All four sites under MESA-ICEMR have been conducting health facility-based surveillance. The GMC has been conducting it from 2012 to current date. The AVBRH, KGVK, and RMRC-NE conducted surveillance between 2014 and 2017. In addition, RMRC-NE has also conducted longitudinal household-based surveys in Karbi Anglong district in Assam (Table 1). The RMRC-NE is also the only MESA-ICEMR site that conducts knowledge, attitude, and practice (KAP) surveys to identify the proportion of survey participants who know the main symptoms, treatment, and preventive measures for malaria. This leads to better community engagement, awareness, and receptiveness to existing malaria control measures. In the same region, surveys on indoor residual spraying (IRS) and insecticide-treated bed net (ITN) usage help assess the extent and effectiveness of the current vector-control regimen.

**Table 1:** Details of data collected in the MESA-ICEMR study sites

Study	Data collected	MESA sites				Clinical impact and translational value
Themes	–	AVBRH	GMC	KGVK	RMRC-NE	
Source of data	a) Routine health information system	•	•	•	•	–
	b) Household-based surveys	–	–	–	•	–
1. Malaria knowledge	1.1 Proportion of population at risk who know the main symptom of malaria	–	–	–	•	1.a Community engagement
	1.2 Proportion of population at risk who know the treatment of malaria	–	–	–	•	1.b Community awareness
	1.3 Proportion of population at risk who know preventive measures for malaria	–	–	–	•	1.c Receptiveness to malaria control measures
2. Vector control	2.1 Number of surveyed subjects sleeping under an ITN or living in house sprayed by IRS in the previous 12 months	–	–	–	•	2.a Reduction in number of infective bites
	2.2 Number of surveyed subjects who slept under an ITN the previous night	–	–	–	•	2.b Reduction in vector habitats
3. Diagnostic testing	3.1 Total number of febrile cases who received a parasitological test for malaria (Annual Blood Examination Rate)	–	•	–	–	3.a Screening capacity in health facilities
	3.2 Improved light microscopy tool for counting low parasitemia *P. vivax* infections	–	•	–	–	3.b Treatment seeking pattern of infected individuals
	–	–	–	–	–	3.c Sensitivity of diagnosis and surveillance
4. Surveillance	4.1 Confirmed malaria cases species wise per 1,000 population at risk	•	•	•	•	4.a Transmission reservoir, intensity, species distribution
	4.2 Number of severe cases species wise	•	•	•	•	4.b Burden of severe cases and species responsible for it
	4.3 Demography—age, gender, and occupation	•	•	•	•	4.c Age, gender occupation as risk factor for infection
	4.4 Travel history	•	•	•	•	4.d Proportion of imported cases
	4.5 Distribution of cases during different seasons	•	•	•	•	4.e Seasonality of cases
	4.6 Spatial and temporal heterogeneity of transmission	–	•	–	–	4.f Stratifying malaria cases
	4.7 Proportion of low density and asymptomatic infections	–	–	–	•	4.g Role of submicroscopic and asymptomatic infections in transmission
	4.8 Genotype of parasites from different study sites	–	•	–	•	4.h Genetic diversity of parasite populations in different regions
5. Treatment	5.1 Proportion of patients with confirmed malaria who received first-line antimalarial treatment according to national policy	•	•	•	•	5.a Heterogenous treatment regimen in India 5.b Transmission-blocking efforts
	5.2 Proportion of patients with *P. vivax* malaria who received radical cure treatment with primaquine	•	•	•	•	5.c Percentage of referred cases from primary healthcare to tertiary health care facility because of severity
	5.3 Proportion of confirmed *P. falciparum* cases who received single, low-dose primaquine	•	•	•	•	5.d Artemisinin resistance status
	5.4 Proportion of severe malaria cases that were referred	–	•	–	–	–
	5.5 Proportion of referred patients with severe malaria that received prereferral treatment	–	•	–		–
	5.6 Proportion of ACT resistant infections (in vitro)	–	•	–	•	–
6. Vector studies	6.1 Geographic and seasonal distribution of *Anopheles* species specific to a site	–	•	–	•	6.a Tailor made control measures depending on vector
	6.2 Time (early/late), place (indoor/ outdoor), and host choice (humans/animals) for biting	–	•	–	•	6.b species 6.c distribution (geographic and seasonal)
	6.3 Insecticide resistance status	–	•	–	•	6.d biting preferences
	6.4 Morphotaxonomic classification of vectors and sibling species analysis	–	•	–	•	6.e vector sensitivity to insecticides used for spraying
	6.5 Sporozoite positivity rate	–	•	–	•	6.f entomological transmission metric
7. Pathogenesis	7.1 Role of invasion ligand/host-receptor interaction	–	•	–	–	7.a Variation in usage of invasion ligands and their association with disease severity
	7.2 Role of *var* gene expression in *P. falciparum* pathogenesis	–	•	–	–	7.b Potential marker of shift from non lethal to lethal form
	7.3 Host cell preference for *P.vivax* infection	–	•	–	–	
	7.4 Short-term *P. vivax* culture system	–	•	–	–	7.c Drug and invasion assays for *P. vivax*
	7.5 Multiplicity of infection	–	•	–	–	7.d Diversity of infection
8. Host Response	8.1 Seroreactivity and antibody response against parasite proteins	–	•	–	–	8.a Biomarkers of population wide-NAI
	8.2 Differential seroreactivity to *P. falciparum* antigens in severe and nonsevere cohorts	–	•	–	–	8.b Variation in immunity
9. Drug resistance	9.1 Monitoring polymorphisms related to drug resistance in the study sites	–	•	–	•	9.a Surveillance of region-specific drug resistance markers
	9.2 Monitoring antimalarial drug sensitivity in vitro	–	•		•	9.b Surveillance of region-specific antimalarial sensitivity
	9.3 Mechanisms of acquiring resistance by parasites against currently used antimalarials and drugs in development	–	•		•	9.c Strategies to avoid drug resistance
10. Impact of control measures						
10A. Prevalence	10A.1 Parasite prevalence: proportion of population with evidence of infection with malaria parasites	•	•	•	•	10.a Changes in transmission reservoir and intensity
10B. Incidence	10B.1 Number and rate of confirmed malaria cases per 1,000 people per year	•	•	•	•	10.b Effectiveness of malaria control regimen in the study sites to curb morbidity and mortality
	10B.2 Number of malaria inpatients	•	•	•	•	
	10B.3 Malaria test positivity rate	•	•	•	•	–
10C. Mortality	10C.1 Number and rate of malaria deaths per 100,000 people per year	•	•	•	•	–
	10C.2 Proportion of inpatient deaths due to malaria	•	•	•	•	–

The clinical and translational value of this data is outlined in the context of malaria control and elimination in India. AVBRH = Acharya Vinoba Bhave Rural Hospital, Wardha; GMC = Goa Medical College, Goa; KGVK = Krishi Gram Vikas Kendra, Ranchi; NAI = Naturally Acquired Immunity; RMRC-NE = Regional Medical Research Center-Northeast, Dibrugarh.

The GMC and RMRC collect data on the total number of febrile patients presenting at these sites who received a parasitological test for malaria. This data informs about the screening capacity in health facilities, treatment seeking pattern of infected individuals, and sensitivity of surveillance. Our observations reported a slide positivity rate (SPR) at 8.4% for GMC, which went up to 10% at the peak of transmission season during the monsoon.^5^ Seasonality of infections is a common theme in all the four MESA-ICEMR sites with the number of infections peaking during the monsoon at their lowest during the dry season and a perennial transmission throughout the year. The proportion of infections caused by *P. falciparum* compared with *P. vivax,* however, varies in each region. The ratio of *P. falciparum:P. vivax* infected cases ratio is highest in Assam and lowest in Goa (Figure 1B). Having a definitive idea about the seasonal peak of transmission in a region aids in tailoring cost-effective vector-control measures, like intensive spraying, before the start of high-transmission season. It also enables vector-control resources to be deployed effectively at the right time and place.

A common questionnaire used at all the sites collects information about age, gender, occupation, and travel history. We noted a higher proportion of cases in the above 5 years age group compared with 5 years and below age group at all the sites. This is characteristic of a low-transmission setting like India. The median age of infection at GMC was 27 years.^5^ All the sites report a higher proportion of infection in male subjects. These statistics reflect the adult male bias of clinical malaria in hypoendemic regions.^6^ A detailed travel history helps in estimating the burden of imported cases, particularly in the urban site of Goa, which sees the highest level of incoming migration in all four sites. This is partly caused by Goa’s status as a tourist hub in India and partly because of the rapidly expanding construction activities in this state,^7^ which draws migrant workers from Eastern India.^5^ In fact, we noted 88.2% of MESA enrolled cases in GMC were born outside Goa and 51.5% of the enrolled cases were construction workers.^5^ Genome-wide analysis of parasite SNPs from these samples will help in determining the origin of infection.^8^ This information regarding local versus imported malaria will be crucial in managing malaria in the urban and periurban transmission settings in Goa that are in the elimination phase.^9^

Treatment data collected by the study sites reflect the heterogeneity of *P. falciparum* malaria treatment regimen in India, which involves different ACTs such as artemether-lumefantrine (AL), artesunate-mefloquine (AS-MQ), and artesunate-sulfadoxine-pyrimethamine (AS-SP) along with primaquine (PQ). Northeast states (Assam, Arunachal Pradesh, and Tripura) and Ranchi used AL + PQ, Wardha used AL + PQ, and AS-SP + PQ, Goa used AS-MQ + PQ (before December 2015) and AS-SP + PQ (post December 2015) for *P. falciparum* malaria treatment. All sites used chloroquine (CQ) and PQ for *P. vivax* malaria treatment. These treatment regimens follow the national guidelines for first line of antimalarial use in India,^10^^,^^11^ which prohibits use of AS-SP in Northeastern India after reports of resistance in that region.^12^ The RMRC and GMC are also involved in in vitro monitoring of artemisinin resistance as detailed in the Antimalarial Resistance section.

The data collected in the MESA-ICEMR sites complement the data collected by National Vector Borne Disease Control Program (NVBDCP) in real time monitoring of the malaria situation and outbreaks in the selected study sites. The MESA-ICEMR data also provide a useful historical record, which can be used to analyze the impact of malaria control measures as well as to improve malaria case management (diagnosis and treatment) in these regions.

## VECTOR STUDIES

Vector-related research in MESA-ICEMR involves monitoring the extent and impact of vector-control methods and entomological surveillance (Table 1). Vector-control measures in India are tailored to transmission intensity settings. This means IRS and long-lasting insecticidal nets (LLINs) are predominantly used in the high-transmission site in Assam and larviciding by biological means is common in the low-transmission setting in Goa.^3^ We noted ITN usage and proportion of the surveyed population living in households that have been sprayed at least once in the last 12 months (Table 1). This in turn, helped us monitor the impact of vector-control measures in our longitudinal household-based survey we conducted in Karbi Anglong, Assam (unpublished).

*Anopheles stephensi*, *An. Culicifacies*, *An. Fluviatilis*, *An. Dirus *(*An. Baimai*), and *An. minimus* are responsible for malaria transmission at the Indian study sites.^13^^,^^14^ Of these, *An. stephensi*, *An. culicifacies* and *An. fluviatilis* have been shown to be responsible for transmission in Goa. We identified *An. subpictus* as the fourth vector for transmission in urban construction sites of Panjim, Candolim, Porvorim, and Margao.^15^ This puts *An. subpictus* as the second species along with *An. stephensi* to be responsible for malaria in urban regions of Goa. Seasonal distribution of these two species indicates that numbers of both the species peaks during rainy months (May–October). However, *An. subpictus* might be the dominant species responsible for transmission in the dry season (November–April), when *An. stephensi* numbers are low.^15^ These two vectors, thus, seem to work in tandem in maintaining urban, year-round transmission in Goa. Of these two, we found *An. stephensi* in Goan survey sites to be resistant to the commonly used insecticides; deltamethrin (8%), Malathion (18%), and DDT (48%)^14^ while resistance status of *An. subpictus* in Goa still needs to be determined.

In a MESA-ICEMR study, we collected *An. stephensi* larvae from construction sites and later grew them under laboratory conditions in an insectary. These larvae were then propagated to establish a pure colony. We have assessed the ability of these laboratory-raised colonized mosquitoes to support *P. vivax* infection from malaria-infected clinical samples collected at GMC and compared with similar ability of wild mosquitoes. The mosquito infection rates and sporozoite load were higher in wild mosquitoes compared with the colonized mosquitoes. The results may reflect the effect of genetic differentiation associated with long-time mosquito colonization on levels of vector susceptibility to *P. vivax.*^16^ We were also able to collect valuable data about vector–parasite interactions in this region with these controlled mosquito-feeding experiments.^17^ No correlation was noted among *P. vivax* parasitemia/gametocytemia with mosquito infection rates but weak correlation was seen with parasite development in the mosquitoes. We also found that a higher mosquito infection intensity in wild *An. stephensi* in this region corresponds to a female to male gametocyte sex ratio close to 1.^17^ Subsequent mosquito-feeding experiments allowed us to explore the optimal conditions for the routine supply of *P. vivax* sporozoites,^18^ which could be used to infect hepatic cell cultures in the future. These experiments will expand our understanding of the latent liver-stage *P. vivax* hypnozoites, but also aid in the design of future point-of-care (POC) hypnozoite diagnosis. We found that optimizing the mosquito rearing method and replacing patient plasma with naïve serum led to more than 2-fold increase in mosquito infection and sporozoite levels.^18^ This finding underscores the importance of monitoring how patient plasma affects mosquito feeding, especially in the regions, where there is a high diversity of human host factors. These established mosquito-feeding procedures give a glimpse of the possible vector–parasite interactions happening in this region but are also testament to the growing MESA-ICEMR capacity for vector studies. The MESA insectary in National Institute of Malaria Research (NIMR) Goa is only 5 km away from GMC and this offers the rare opportunity of mosquito-feeding experiments with straight from the arm clinical samples within 45 minutes of blood collection. The optimization of sporozoite production has high potential, not just for generating a ready resource for liver-stage research facilitating the development of antirelapse interventions, but also for other future studies on transmission-blocking immunity and vaccines.

## ANTIMALARIAL RESISTANCE

Overall, malaria cases have been declining in India since 1996, however, *P. falciparum* prevalence remains steady.^19^ Widespread drug resistance to ACTs would alter the trajectory and goal of eliminating malaria in India by 2030. The National Antimalarial Drug Resistance Monitoring System was established in 2009 to continuously monitor sentinel sites throughout India using a combination of molecular biology and clinical approaches.^20^ Continued multifaceted approaches for resistance detection are vital to India’s efforts to control malaria. Academic-based research investments, like the MESA-ICEMR, play an important role in supplementing resistance surveillance efforts. The MESA-ICEMR laboratories were the first to conduct and combine ring-stage survival assays (RSAs) with genotypic characterization to identify reduced sensitivity to artemisinin in parasites from Northeast and Southwest India.^21^

We demonstrated using RSAs that a relatively small patient set (*N* = 22) from Northeast India (Assam, Arunachal Pradesh, and Tripura) and Goa had parasites with elevated tolerances to dihydroartemisinin.^21^ In this study, a single isolate out of 22 displayed the A675V nonsynonymous mutation in *Pfkelch13*, which previously had been declared by the World Health Organization (WHO) as a candidate marker for artemisinin resistance.^22^

The four confirmed (in vitro and in vivo) artemisinin resistance *Pfkelch13*mutations (Y493H, R539T, I543T, and C580Y) from Southeast Asia have not been found in India yet. It is unclear which mutations originate from founder populations within India and which are imported from its neighbors. We evaluated the intrinsic capacity of Indian parasites to acquire in vitro resistance to novel inhibitors and their rates of mutagenesis are comparable to those from Southeast Asia (manuscript in progress). Although the capacity to mutate genomes may be similar, South Asia may still be in a good position to minimize the development and spread of artemisinin resistance by vigilant molecular and clinical surveillance. The MESA-ICEMR collection sites spread throughout India have the capacity to identify founder and/or imported resistant populations.

Choice of partner drugs included in ACTs is critical to preserving artemisinin’s ability to serve as a frontline antimalarial. Although Northeast India relies on the artemether–lumefantrine combination, most of India is using artesunate-sulfadoxine-pyrimethamine to care for uncomplicated malaria cases.^11^ A MESA-ICEMR study of 10 culture-adapted isolates (2012–2013) from Goa discovered that antifolate resistance genotypes were not predictive of phenotype (manuscript in preparation). Parasites with two mutations in *Pfdhfr* (59R + 108N) and a single *Pfdhps* mutation (437G), or none, were phenotypically as resistant as triple mutants for both genes (unpublished). We suspect that folate salvage was the primary modulator of antifolate resistance levels in the Goan parasites. Although rapid parasite clearance is dependent on artemisinins quickly reducing biomass, the day 3 positivity and late-treatment failures point to possible preexisting antifolate resistance genotypes.^12^^,^^23^^–^^25^ The findings make it clear that artemisinin, sooner than later, will have to be partnered with compounds for which there is much less circulating, preexisting resistance in India.^26^ In the case of antifolate resistance, phenotypic and genotypic surveillance activities will have to be increased to better inform policy-makers. The MESA-ICEMR will continue to monitor resistance to artemisinin and partner drugs at the genotypic and phenotypic levels.

## PATHOGENESIS AND HOST RESPONSE

The MESA-ICEMR team has collected data on around 600 patients hospitalized with severe malaria (SM) and about 2,000 uncomplicated malaria patients, so far (unpublished data). Clinical assessment of the degree of malaria severity was done in all sites. Apart from estimating the burden of SM, we use laboratory-based data from severe and uncomplicated patient samples to explore different facets of the complex pathogenesis involving several intricate processes in the malaria parasite and host (Table 1).

Steady, year-round access to infected blood from *P. vivax* patients at GMC has allowed the development of tools to overcome experimental shortcomings with the study of *P. vivax*. First, the parasitemia of *P. vivax* infections are usually low within patients and we have improved counting methodologies for low parasitemia infections by light microscopy.^27^ Investigations into *P. vivax* biology are severely limited by the lack of a continuous in vitro culture system. Access to a steady collection of *P. vivax* patient isolates has facilitated significant advances in our ability to short-term culture *P. vivax*. We have discovered that short-term culturing *P. vivax* in a hematopoietic stem cell medium permits highly efficient maturation of parasites from the ring to schizont stage of the intraerythrocytic developmental cycle (IDC) of the parasite.^28^ This has allowed us to establish robust *P. vivax* drug and invasion assays using both frozen parasites as well as fresh clinical isolates from GMC. This, in turn, has set the stage for exploring the molecular basis of *P. vivax* invasion, host cell tropism, and pathogenesis. The MESA-ICEMR has shown that gametocytes from cryopreserved patient *P. vivax* isolates are stable for use in mosquito infection.^29^ This finding, combined with our ability to short-term culture *P. vivax* and the high frequency of gametocytes that we observe in clinical isolates at GMC, will be important resources for future transmission studies.

*P. vivax* invasion of red blood cells is an obligatory step in the parasite life-cycle but there is a marked deficiency in our understanding of the invasion process in *P. vivax*, again primarily due to the lack of a continuous in vitro culture system for this parasite. *Plasmodium vivax* is unique due to its strong preference for invading reticulocytes, the youngest red blood cells.^30^^,^^31^ Using the large number of isolates collected from the GMC, we have shown that the patient isolates exhibit variation in reticulocyte preference.^32^ We have also developed a flow cytometry-based osmotic lysis assay to demonstrate changes in reticulocyte stability during *P. vivax* infection.^33^

The variation in the usage of *P. vivax* invasion ligands, the existence of discrete invasion pathways, and their association with disease severity are poorly understood. The PvDBP/DARC interaction is thought to be essential for *P. vivax* invasion^34^ and the lack of DARC on red blood cells of people in Sub-Saharan Africa is likely to have been selected for by *P. vivax*.^34^^,^^35^ However, there is increasing evidence of *P. vivax* infection in DARC-negative individuals^36^^–^^42^ that could have profound public health implications for the spread of this parasite. A report of PvDBP duplication^43^ in field isolates in Madagascar, Sudan, and Cambodia may present a possible mechanism for invasion of DARC-negative red blood cells. We, therefore, tested for PvDBP duplication within isolates collected at GMC, but found that they are absent in *P. vivax* isolates from India, suggestive of greater geographical variation in *P. vivax* parasites.^44^ In future, we plan to test whether there is any protective effect of DARC genotypes by determining the DARC genotypes and *P. vivax* parasite burdens in malaria patients at the GMC.

Our *P. vivax* invasion assay has also allowed us to investigate *P. vivax* invasion ligand gene expression and variations in invasion. In addition to the PvDBP/DARC invasion pathway, the MESA-ICEMR team has also investigated the newly identified reticulocyte-tropic invasion pathway between the *P. vivax* RBP2b invasion ligand and host transferrin receptor (TfR1).^45^ We measured invasion inhibition in clinical *P. vivax* isolates using host-targeted small molecules/antibodies (anti-DARC and anti-TfR1) and we observed that while invasion could be inhibited in all strains, there was significant variation in usage of each invasion pathway.^46^ Furthermore, we observed that combinatorial inhibition using both anti-DARC and anti-TfR1 inhibitors led to inhibition synergy, which may have future implications for *P. vivax* vaccine development.^46^

Our work in *P. falciparum* pathogenesis has involved *var* gene (*PfEMP1*) profiling in complicated and uncomplicated malaria patients from Goa.^47^^,^^48^ We carried out these studies to understand the crucial role of differential expression of *var* genes in pathogenesis of SM through infected RBC (iRBC) sequestration and the role of parasite biomass.^47^ About 77% of the SM study patients presented more than one severity criterion, as set by the WHO, while 57% showed more than three different severity criteria, indicating multisystem disorder. We demonstrated that SM patients showed higher parasite biomass than uncomplicated malaria patients as indicated by plasma PfHRP-2 levels—the surrogate biomarker assigned for total parasite biomass.^47^ Quantitative RT-PCR (qRT-PCR) analysis showed that type A *var* transcripts were overrepresented in SM than the type B and C *var* transcripts. Group A *var* variants, specifically DC8 and DC6 PfEMP-1 were previously found to be associated with pediatric SM.^49^ This subset of PfEMP-1 includes mediators responsible for “rosetting” and endothelial protein C receptor (EPCR) binding, two distinct erythrocyte adhesion categories known to cause SM.^50^^–^^52^ Higher levels of serum PfHRP-2 positively correlated with elevated transcript levels of EPCR binding (DC8 and DC6) and rosetting (DC5) *var* phenotypes in SM patients. Our data suggests that specific types of PfEMP-1 may promote higher parasite biomass, ultimately contributing to severe pathophysiological outcomes. Machine learning analysis indicated the combination of high parasite biomass (assessed through PfHRP-2 levels) along with DC6 and DC8 encoding *var* transcripts was the strongest indicator of severity and hospitalization in the study subjects from Goa.^47^ In vitro binding studies showed moderate inhibition of EPCR-APC (Activated Protein C) binding using DC8 CIDRα from SM patients, further suggesting that this differential binding might play a role in severe manifestation of the disease in low-transmission settings like India. Molecular analysis of EPCR-APC blockade by PfEMP1 domains further supports this phenomenon.^48^

Our subsequent meta-analysis study involving three different cohorts with pediatric malaria in Tanzania and Malawi, and adult malaria in Goa, indicated that parasite biomass and *var* gene expression profiles play independent and complementary roles in development of SM, not only in African children, but also in Indian adults.^49^ Additionally, even when the *var* gene expression profiles show variation within host and among sites, the *var* gene profile signatures associated with SM were highly similar across three cohorts included in the study. There are differences in disease presentation in African children and Indian adults, which is to be expected owing to differences in transmission intensity in African and Indian regions. Despite these differences, SM in African children and Indian adults is definitively linked to increased transcription of *var* variants predicted to bind EPCR.^49^ Further, this meta-analysis^49^ suggests a strong role of parasite biomass in development of SM and indicates that sequestering parasite populations might increase endothelial cell activation and microvascular obstruction.^53^^–^^56^ This meta-analysis is consistent with previous studies, where DC8 and group A *var* transcripts were implicated in SM.^52^^,^^57^

The interaction/s of malaria parasites with the host that are responsible for clinical outcome vary with parasite biology and host physiology. Naturally acquired immunity (NAI) of the human host to parasites play an important role in malaria pathogenesis. Development of NAI depends on duration and degree of exposure to the parasites^58^^,^^59^ and can vary significantly in different endemic areas due to variation in degree of transmission and specificity toward infecting *Plasmodium* species.^60^ In India, malaria transmission is influenced by many factors such as changing seasons, the existence of multiple *Plasmodium* species and their vectors, and diverse epidemiological profiles that can directly contribute to the development of NAI. Seroreactivity and antibody response against parasite proteins are useful biomarkers of population-wide NAI, and significantly different levels of these markers had previously been observed at different Indian sites.^61^ The MESA-ICEMR has optimized the methods to identify seroreactive protein markers using protein arrays in both uncomplicated and complicated patient sera.^62^ Seroreactivity and antibody response from patients enrolled in Goa were studied with protein microarray chips that display 500 P. *falciparum* and 515 *P. vivax* protein respectively.^63^ *Plasmodium falciparum* patient Sera showed a broader immune response than *P. vivax* patient Sera. This observation in India is consistent with previous ICEMR protein array data from Kenya.^64^ Our study identified five *P. falciparum* and two *P. vivax* antigens with differential seroreactivity among inpatients and outpatients. Severe *falciparum* and *vivax* malaria patients showed significantly higher antibody response than uncomplicated patients. Interestingly, several conserved proteins like MSP, PHIST, and NOT family proteins were found to be seroreactive only in nonsevere patients in both falciparum as well as vivax infections. Altogether, 248 *P. falciparum* and 73 *P. vivax* seroreactive proteins were identified in the MESA-ICEMR study, which is consistent with similar studies published earlier.^64^^–^^68^ Although the majority of these putative antigen proteins are predicted to be either exported to the iRBC surface or present on the merozoite surface, some are expected to be nuclear, cytoskeletal, and cytoplasmic. Immunity patterns from the high-transmission endemic areas across age groups differ from the immunity patterns from low-transmission areas. In Africa, adults develop immunity that protects against SM by repeated exposure throughout childhood. Our seroanalysis study provides insights into antibody-dependent immune responses against malaria in low-transmission settings (India). Here, malaria in adults is more likely to evolve into severe disease due to lack of repeated exposures to the parasites during childhood.^69^ The MESA-ICEMR data provides the first evidence of differential seroreactivity to *P. falciparum* antigens in severe versus nonsevere cohorts,^62^ and complements the efforts undertaken by other ICEMR programs to compare the variation in immunity against malaria.^64^

## CONCLUSION

**Figure 2.**
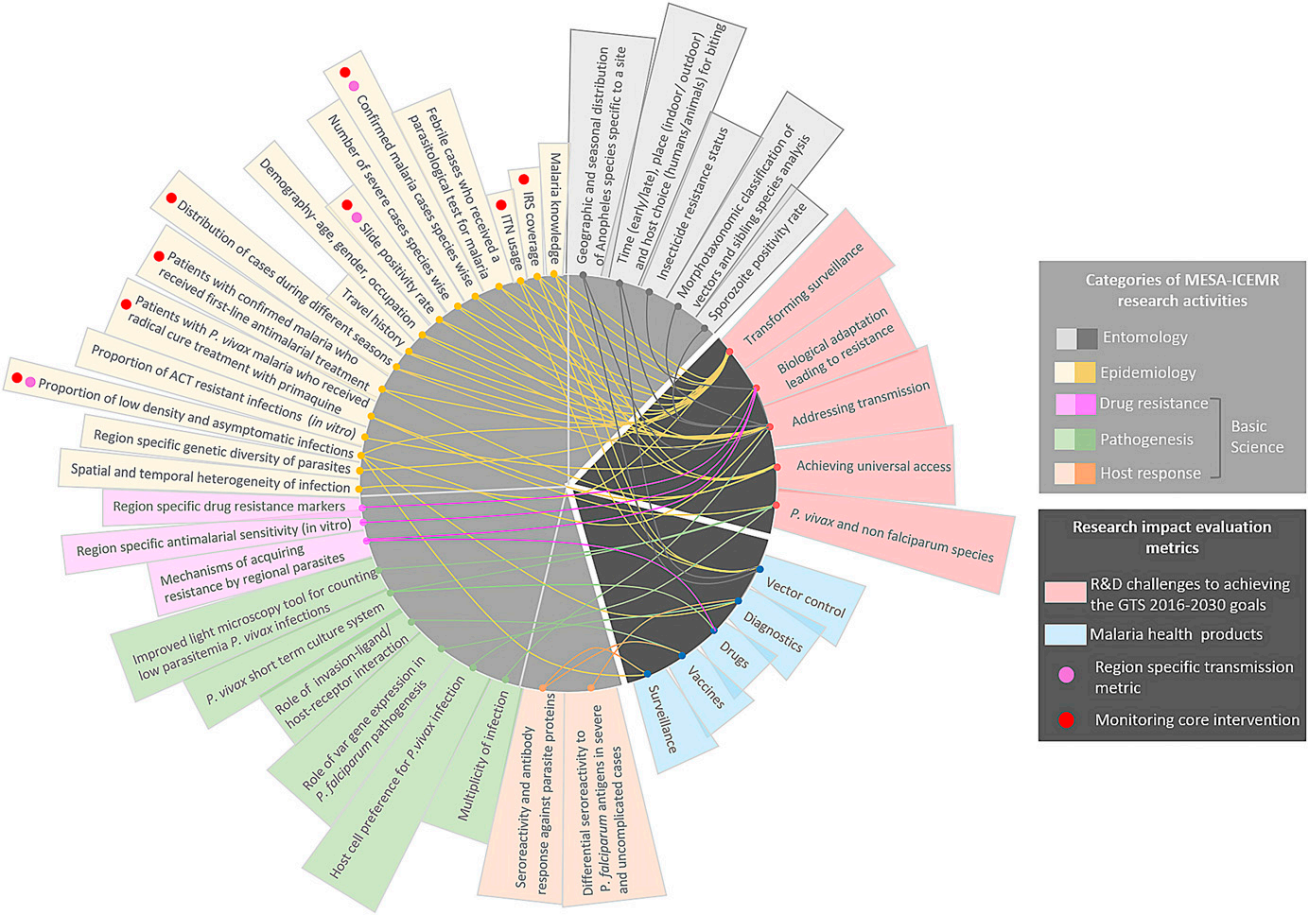
The multidisciplinary integrated research of MESA-ICEMR is capable of delivering on the research and development challenges identified by the WHO in achieving GTS 2016–2030 goals. The MESA-ICEMR basic sciences research with clinical samples can lead to improvement of current malaria health products or lead to the development of novel products in these categories.

Malaria accounted for 5 million cases in India in 2020.^70^ India has reported a 78% decrease in the number of malaria cases from the approximately 200 million cases in 2000.^70^ Efforts from government, nongovernment and private sectors have played a significant role in achieving this feat.^71^^,^^72^ The integrated multidisciplinary research approach of the MESA-ICEMR plays a complementary role in describing malaria in India. Together with national and subnational malaria stakeholders at the MESA-ICEMR study sites, joint efforts are aligned with the goal of malaria elimination in these regions as envisioned by the WHO Global Technical Strategy (GTS) for Malaria 2016–2030,^73^ NFME in India 2016–2030,^2^ and the NSP 2017–2022^3^ (Figure 2). Key MESA-ICEMR contributions include collection and dissemination of health facilities data and survey-based data on malaria in government as well as public/private healthcare facilities. The experience of MESA-ICEMR in real time monitoring of clinical malaria cases through databases like RedCap and ClinEpiDB is important for emerging national digital healthcare surveillance.^74^ Our household-based surveys provide data on vector-control coverage and KAP indicators. The surveillance data monitors the transmission metrics in these regions and assesses the impact of interventions (IRS, LLINs, RDTs, and ACTs) on malaria prevalence, incidence, and mortality. This information could help in designing a combination of interventions tailored to local contexts, particularly in the very low-transmission regions in India like Goa. Our entomological surveillance in Assam and in Goa give valuable information about vector species responsible for malaria transmission as well as vector behavior and their insecticide resistance status in these study sites. Our model of determining imported cases through detailed travel history and SNP analysis in Goa could be very useful in similar low-burden Category 1 Indian states in the elimination phase. Adequate case management, involving diagnosis, and treatment is one of the key interventions identified in GTS and NFME. The MESA-ICEMR aims to understand the region-specific molecular variations in host cell invasion and cytoadherence that may precipitate SM at the study sites, along with potential loss of diagnostic tools due to *Pfhrp-2* deletions. We also monitor the current status of ACT sensitivity in the Indian parasites from these sites. Together, these efforts put the MESA-ICEMR team in a competent position for complementing and informing diagnosis and treatment regimens in this region. Moreover, the MESA-ICEMR has built strong research facilities at the study sites, particularly Assam and Goa. This makes us uniquely poised to help deliver on the malaria research and development needs outlined by the WHO^75^ and to test the impact of region-specific malaria control measures.
